# Oxytetracycline on the Wetland Macrophyte *Bidens laevis* L. Seedlings: Uptake, Bioaccumulation, Translocation and Biochemical Stress Responses

**DOI:** 10.3390/plants15182741

**Published:** 2026-09-08

**Authors:** Daniela M. Truchet, Débora J. Pérez, Diana M. Villagran, Fernando G. Iturburu, Julieta R. Mendieta, Sandra K. Medici, Mirta L. Menone

**Affiliations:** 1 Instituto de Investigaciones Marinas y Costeras (IIMyC, CONICET-UNMDP), Consejo Nacional de Investigaciones Científicas y Técnicas (CONICET), Facultad de Ciencias Exactas y Naturales, Universidad Nacional de Mar del Plata (UNMDP), Dean Funes 3350, Mar del Plata 7600, Buenos Aires, Argentina; dianavillagran88@gmail.com (D.M.V.); fernando.g.iturburu@gmail.com (F.G.I.); mirta.menone@gmail.com (M.L.M.); 2 UMR CNRS 6553 Ecosystems, Biodiversity, Evolution (ECOBIO), University of Rennes, 35000 Rennes, France; 3 Departamento de Producción Vegetal, Suelos e Ingeniería Rural, Consejo Nacional de Investigaciones Científicas y Técnicas (CONICET), Facultad de Cs. Agrarias, Universidad Nacional de Mar del Plata (UNMDP), Ruta Nacional 226 km 73.5, Balcarce 7620, Buenos Aires, Argentina; deborajesabelperez1981@gmail.com; 4 Facultad de Ingeniería, Universidad de la Fraternidad de Agrupaciones de Santo Tomás de Aquino, Gascón 3145, Mar del Plata 7600, Buenos Aires, Argentina; 5 Instituto de Investigaciones Biológicas (IIB), Consejo Nacional de Investigaciones Científicas y Técnicas (CONICET), Universidad Nacional de Mar del Plata (UNMDP), Dean Funes 3250, Mar del Plata 7600, Buenos Aires, Argentina; julietamendieta@hotmail.com; 6 Comisión de Investigaciones Científicas de La Provincia de Buenos Aires (CIC), Calle 526 entre 10 y 11, La Plata 1900, Buenos Aires, Argentina; 7 Instituto de Investigaciones en Producción, Sanidad y Ambiente (IIPROSAM), Consejo Nacional de Investigaciones Científicas y Técnicas (CONICET), Facultad de Ciencias Exactas y Naturales, Universidad Nacional de Mar del Plata (UNMDP), Dean Funes 3350, Mar del Plata 7600, Buenos Aires, Argentina; smedici@farestaie.com.ar; 8 Fares Taie Instituto de Análisis, Magallanes 3019, Mar del Plata 7600, Buenos Aires, Argentina

**Keywords:** macrophytes, antibiotics, tetracyclines, translocation, biomarkers, IBRv2

## Abstract

Oxytetracycline (OTC) is a widely used veterinary antibiotic worldwide due to its extensive use in livestock production. However, information regarding its uptake, biochemical and physiological effects in macrophytes remains limited. This study evaluated OTC uptake, bioaccumulation, translocation, and associated biochemical and physiological responses in seedlings of the wetland macrophyte *Bidens laevis*, exposed for 48 h to environmentally relevant concentrations (0, 0.05, 0.5, 5, 10 and 100 µg L^−1^). OTC accumulated in the roots and shoots, with bioaccumulation factors exceeding 1 in all treatments. At low concentrations, OTC was translocated to the shoots, whereas root retention predominated at 100 µg L^−1^. Antioxidant enzyme activities in shoots generally decreased with increasing OTC concentrations, while root responses remained comparatively stable. Chlorophyll contents increased with exposure, whereas lipid peroxidation showed limited variation. Multivariate analyses revealed contrasting effects of exposure concentration and organ accumulation on plant responses. Overall, *B. laevis* exhibited a high capacity to accumulate OTC and tolerate environmentally relevant concentrations, supporting its potential use in phytoremediation strategies.

## 1. Introduction

Freshwater ecosystems are increasingly threatened by emerging contaminants, particularly pharmaceuticals, whose production, consumption, and disposal have grown substantially over recent decades [1]. Pharmaceuticals and personal care products (PPCPs) comprise a diverse group of compounds used for therapeutic, prophylactic, and cosmetic purposes in both human and veterinary medicine [2]. Their continuous release into the environment through wastewater effluents, agricultural runoff, industrial discharges, and improper disposal practices has contributed to the widespread contamination of surface waters. Because conventional wastewater treatment plants are often unable to completely remove these compounds, pharmaceuticals are frequently detected in aquatic ecosystems worldwide [3].

Veterinary pharmaceuticals are widely used worldwide to maintain animal health and support livestock production, with global consumption estimated to exceed 100,000 tons annually [4]. Their use is of particular concern in the context of the intensification of livestock production required to meet the growing global demand for animal-derived products, especially in developing and emerging economies [5]. However, patterns of pharmaceutical use vary considerably among countries according to their production systems, regulations, and economic activities [6]. In Europe, for example, penicillins accounted for 32.7% of total veterinary antimicrobial sales in 2022, followed by tetracyclines (23.5%) and sulfonamides (9.4%) [3].

The environmental occurrence and fate of veterinary pharmaceuticals depend strongly on their physicochemical properties. Following administration, a substantial fraction of these compounds is excreted unmetabolized and can subsequently enter aquatic environments through manure application, runoff, wastewater discharges, and livestock-associated activities [7]. For instance, the highly lipophilic antiparasitic ivermectin is mainly excreted in feces, facilitating its transfer to soils, sediments, and aquatic food webs [8]. Similarly, antibiotic contamination has been linked to cattle production systems, including feedlots, concentrated animal feeding operations (CAFOs), dairy farms, slaughterhouses, and free-range livestock. In some regions, effluents from these activities may be discharged directly or indirectly into rivers and streams, constituting an important source of pharmaceutical inputs to freshwater ecosystems [9]. This might also include antimicrobial-resistant bacteria and the resistance genes that emerge and are excreted through feces [10].

Among the veterinary pharmaceuticals, oxytetracycline (OTC) is one of the most widely used antibiotics worldwide [6]. OTC is a broad-spectrum tetracycline produced by the actinomycete *Streptomyces rimosus* and is commonly employed in veterinary medicine for the prevention and treatment of infections caused by both Gram-positive and Gram-negative bacteria. Its antibacterial activity is based on the inhibition of protein synthesis through binding to the 30S ribosomal subunit, thereby preventing the attachment of aminoacyl-transfer RNA (tRNA) to the ribosome–mRNA complex and disrupting peptide elongation [11]. At elevated concentrations, tetracyclines may interfere with protein synthesis in eukaryotic cells; however, their toxicity is considerably lower because mammalian cells lack the specialized transport systems present in bacteria and possess ribosomes with lower sensitivity to these compounds. Consequently, tetracyclines exhibit a high degree of selective toxicity toward bacterial organisms [11]. Due to its extensive use in livestock production and aquaculture [12], OTC is frequently detected in environmental matrices, including surface waters, sediments, soils, and wastewater effluents and biota [12].

Several technologies have been developed to mitigate pharmaceutical contamination in wastewater, including intensive biological processes such as activated sludge systems, membrane bioreactors, and anaerobic digestion, as well as extensive or nature-based solutions, including stabilization ponds and constructed wetlands [13]. Among these alternatives, constructed wetlands have received increasing attention because they provide an environmentally sustainable and cost-effective approach for removing contaminants from aquatic systems. In these systems, macrophytes play a key role by facilitating contaminant uptake, transformation, and retention. Several studies have shown that aquatic plants can bioaccumulate pharmaceuticals at environmentally relevant concentrations without exhibiting severe phytotoxic effects [14]. Furthermore, the biomass generated during phytoremediation may be valorized through anaerobic digestion for biogas production, contributing to a circular and environmentally sustainable economy [15,16].

Previous studies investigating the effects of OTC on aquatic macrophytes have reported contrasting results depending on species and exposure conditions. High OTC concentrations (>10 mg L^−1^) inhibited the growth of the submerged macrophyte *Vallisneria natans* [17]. Similarly, growth inhibition was reported for *Egeria densa* and *Ceratophyllum demersum* exposed to 250 μg L^−1^ OTC in nutrient-enriched conditions [18]. In contrast, lower concentrations (<25 μg L^−1^) did not induce significant physiological damage in *E. densa*, *Azolla caroliniana*, or *Taxiphyllum barbieri* after 24 h of exposure [19]. More integrative assessments have demonstrated that OTC can impair plant growth, disrupt mitochondrial metabolism, and induce oxidative stress in *Lemna minor* [20]. These findings suggest that the effects of OTC on aquatic plants are highly species-dependent and may vary according to exposure concentration and duration.

Despite the growing number of studies addressing pharmaceutical contamination in aquatic environments, ecotoxicological research on macrophytes has focused primarily on a limited number of model species, such as those from the genus *Lemna* spp., *Myriophyllum* spp., and *Hydrilla* spp. [21]. Consequently, information regarding the responses of native South American macrophytes remains scarce. *Bidens laevis* L. (Asteraceae) is a perennial emergent macrophyte widely distributed in wetlands and marshes of the Pampas region of Argentina associated with agriculture and livestock impacts. Previous studies have shown that this species is sensitive to several pesticides, as evidenced by alterations in genotoxicity and oxidative stress biomarkers [22,23,24]. However, it has also demonstrated tolerance to other contaminants, including chlorpyrifos [25] and the veterinary antibiotic monensin [26], highlighting its potential as a candidate species for phytoremediation applications.

Given the increasing interest in macrophytes as nature-based solutions for mitigating contaminants in aquatic ecosystems [21], the aim of this study was to comprehensively evaluate the uptake, bioaccumulation, and translocation of OTC in *B. laevis*. In addition, the biochemical and physiological responses associated with OTC exposure were assessed through the determination of antioxidant biomarkers (catalase -CAT-, superoxide dismutase -SOD-, glutathione-s-transferase -GST-, guaiacol peroxidase -GPOD-), reactive oxygen species (hydrogen peroxide -H_2_O_2_-), oxidative damage (malondialdehyde -MDA-) and photosynthetic pigments. To our knowledge, this is the first study integrating OTC accumulation dynamics with oxidative stress and photosynthetic responses in a native South American macrophyte exposed to environmentally relevant concentrations (0.05, 0.5, 5, 10, and 100 μg L^−1^). Thus, the findings of this study advance our understanding of the tolerance of *B. laevis* to OTC and support its potential application in the mitigation and phytoremediation of livestock effluents contaminated with veterinary antibiotics.

## 2. Results

### 2.1. OTC in Plants: Mass, Bioaccumulation, Translocation and Estimations for Recovery

Table 1 summarizes the OTC concentrations, accumulated mass, and distribution percentage between the shoots and roots of *B. laevis* after 48 h of exposure. In the shoots, OTC concentrations ranged from 77.3 (±65.4) to 165.3 (±8.08) µg kg^−1^ fw. No significant differences were detected among most treatments, although the highest exposure concentration (100 µg L^−1^) exhibited significantly higher shoot concentrations than the lower treatment (0.5 µg L^−1^). In the roots, OTC concentration ranged from 32 (±22.6) to 931 (±210.7) µg kg^−1^, and significant differences were detected between the 100 µg L^−1^ treatment and the other treatments. Likewise, no significant differences were observed between the shoot and root concentrations within individual treatments. When considering the whole plant, the total OTC concentrations ranged from 74.4 (±43.3) to 471.6 (±432.4) µg kg^−1^, and were significantly higher in the 100 µg L^−1^ treatment than in the remaining exposure groups.

Regarding accumulated mass, shoot OTC mass did not differ significantly among treatments. In contrast, root OTC mass was significantly higher in the 100 µg L^−1^ treatment than in all other treatments. A similar pattern was observed for total plant OTC mass, with the highest values recorded at 100 µg L^−1^. No significant differences in accumulated OTC mass were detected between the roots and shoots within the same treatment.

The distribution of OTC between organs varied with exposure concentration. At 0.05 µg L^−1^, most of the accumulated OTC was found in the shoots (84.4%), whereas only 15.6% was retained in the roots. At intermediate concentrations (0.5–10 µg L^−1^), OTC was distributed relatively evenly between the shoots and roots, with each organ accounting for approximately 50% of the total accumulated mass. In contrast, at 100 µg L^−1^, OTC accumulation was strongly concentrated in the roots, which accounted for 82.2% of the total OTC recovered in the plant.

BAFs (Table 2) were greater than 1 in all treatments and organs, indicating that *B. laevis* accumulated OTC from the exposure medium under all tested concentrations. However, BAF values decreased with increasing OTC concentrations, with the highest values recorded in the lowest exposure treatments, suggesting a greater root uptake and shoot translocation efficiency at low environmentally relevant concentrations.

**Figure 1:**
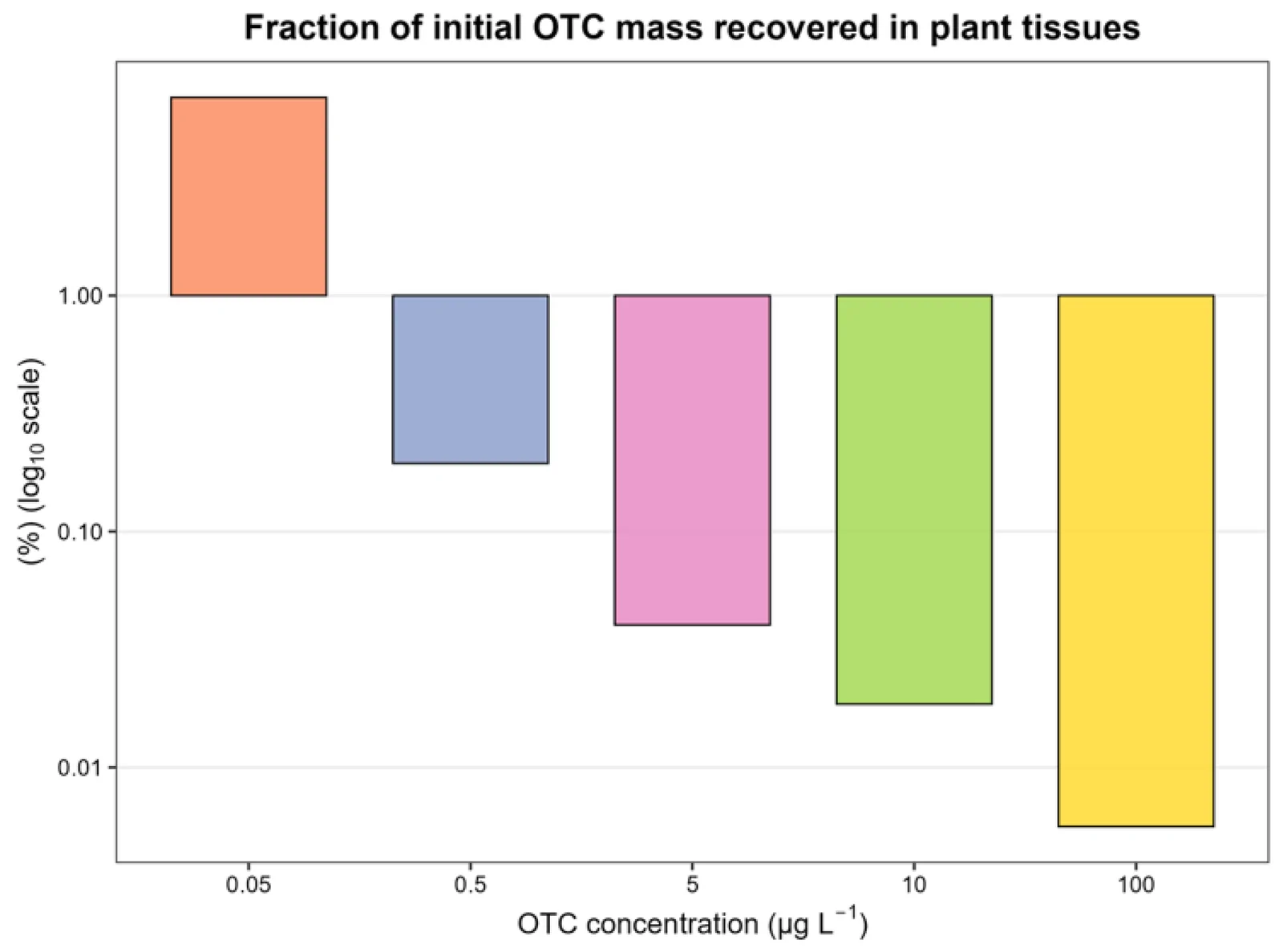
Estimated fraction of the initial OTC mass recovered in *B. laevis* tissues after 48 h of exposure to different OTC concentrations (0.05–100 µg L^−1^). The y-axis is presented on a logarithmic (log_10_) scale to facilitate visualization across treatments.

TFs exceeded 1 only in the 0.05 µg L^−1^ treatment, indicating the efficient transport of OTC from the roots to shoots under low-exposure conditions. In contrast, TF values below 1 at higher concentrations revealed a progressive retention of OTC in the root tissues, particularly at 100 µg L^−1^, where most of the accumulated antibiotic remained belowground. This pattern is consistent with the estimated recovery of OTC in plant tissues (Figure 1). The proportion of the initial OTC mass recovered in plant biomass was highest at the lowest exposure concentrations and progressively decreased with increasing OTC levels in the exposure medium.

### 2.2. Effects of OTC in Biochemical and Physiological Biomarkers and IBRv2

**Figure 2:**
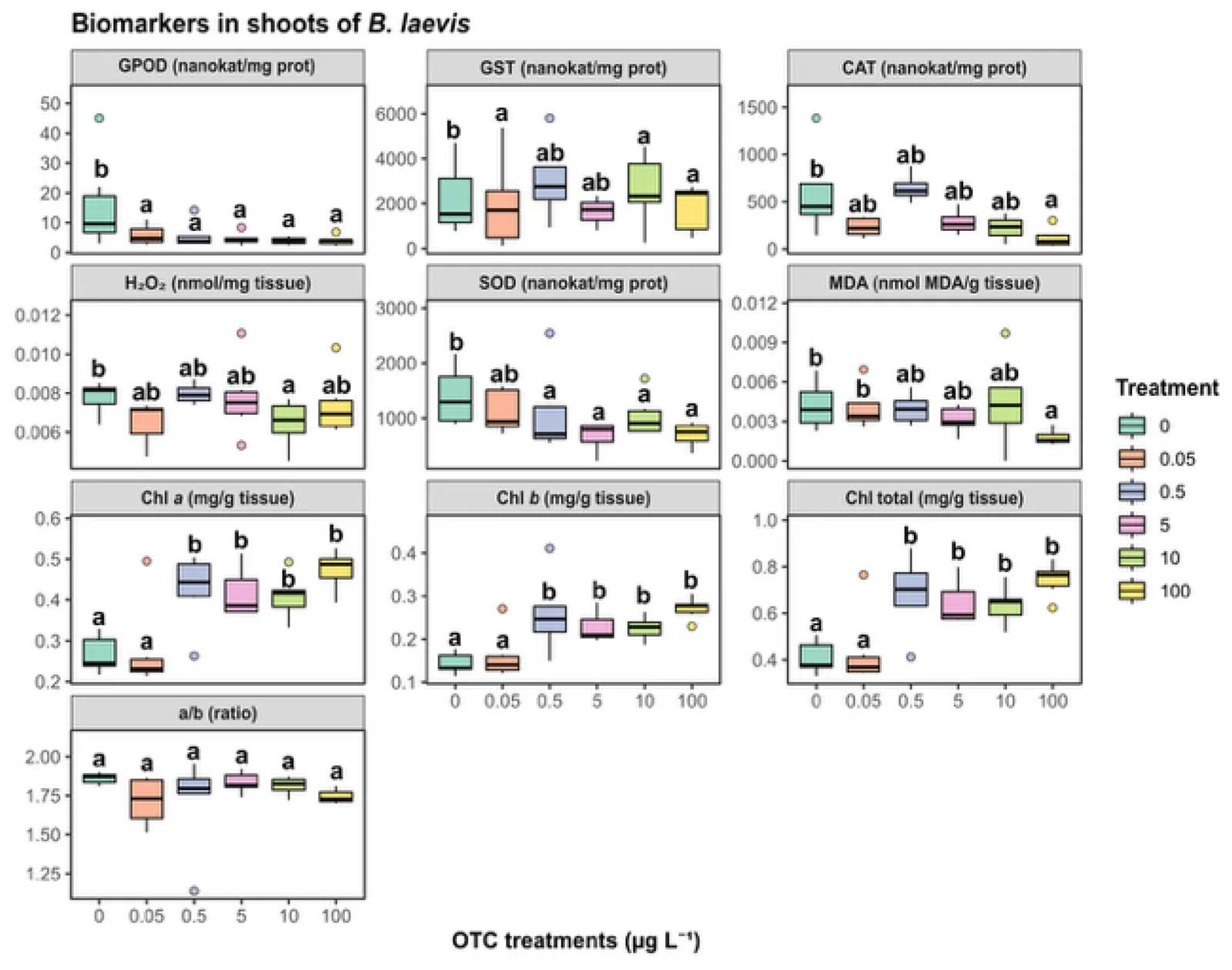
Oxidative stress (GPOD, GST, CAT, H_2_O_2_, SOD, and MDA) biomarkers in the shoots and physiological parameters in leaves (Chl *a*, Chl *b*, Chl total and a/b) of *B. laevis* across all treatments. Similar lowercases indicate no significant differences between the treatments (one-way ANOVA, Tukey post hoc, *p* ≥ 0.05).

For the shoot biomarkers (Figure 2), GPOD, CAT, and SOD activities generally decreased with increasing OTC concentrations, with significant inhibition observed between the control and the highest OTC treatment. The H_2_O_2_ levels did not differ significantly among treatments, except at 10 µg L^−1^, where the lowest values were recorded compared to the control. Similarly, MDA concentrations remained comparable to the control in most treatments, although the highest OTC concentration resulted in significantly lower values than those observed in the control and 0.05 µg L^−1^ treatments. In contrast, chlorophyll-related parameters generally increased with increasing OTC concentrations, whereas the Chl *a*/Chl *b* ratio remained unchanged across treatments.

**Figure 3:**
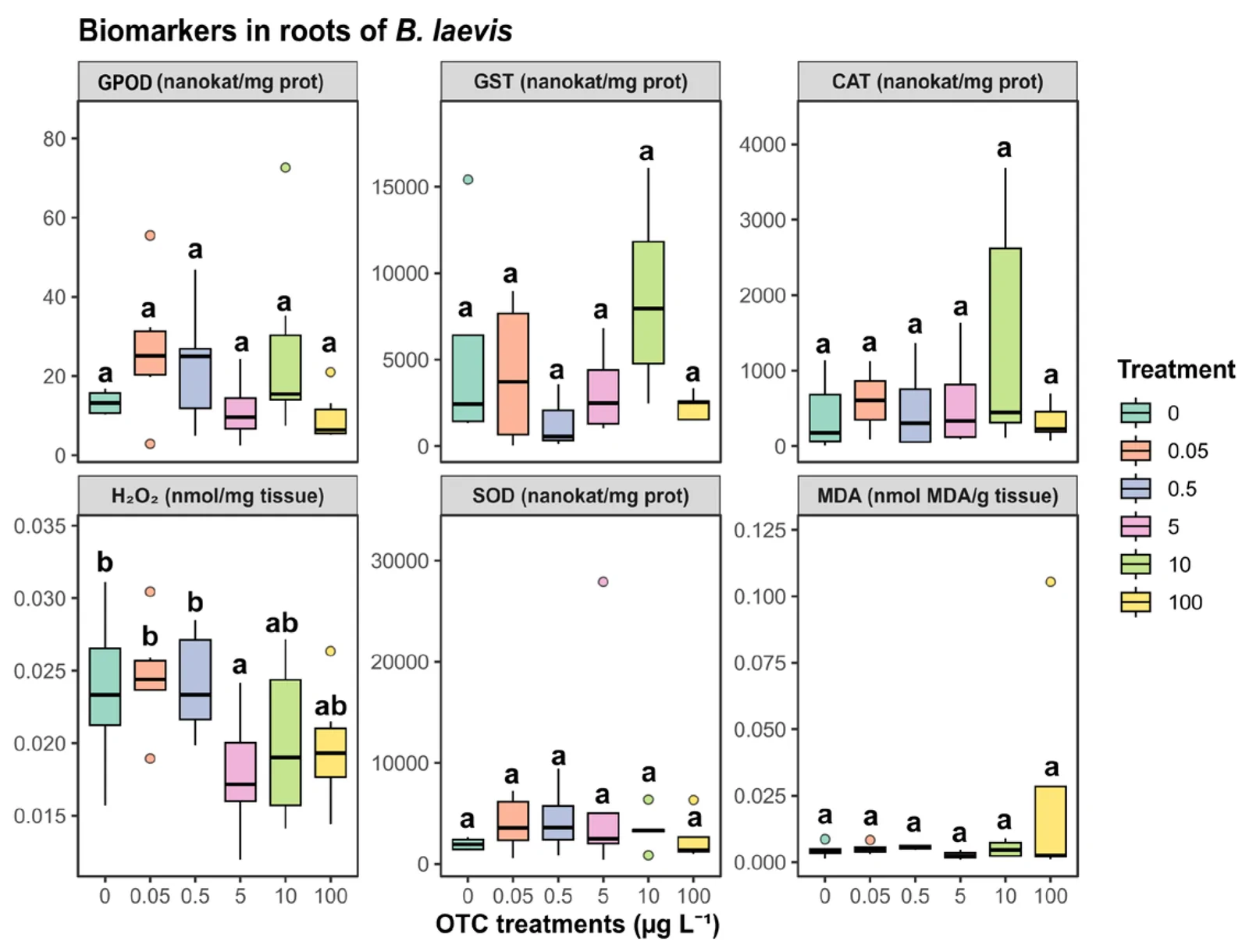
Oxidative stress (GPOD, GST, CAT, H_2_O_2_, SOD, and MDA) biomarkers in the shoots of *B. laevis* across all treatments. Similar lowercases indicate no significant differences between the treatments (one-way ANOVA, Tukey post hoc, *p* ≥ 0.05).

In the roots (Figure 3), GPOD, GST, CAT, SOD, and MDA did not show significant treatment-related differences. The H_2_O_2_ levels were highest in the control, 0.05, and 0.5 µg L^−1^ treatments, differing significantly only from the 5 µg L^−1^ treatment, which exhibited the lowest values.

**Figure 4:**
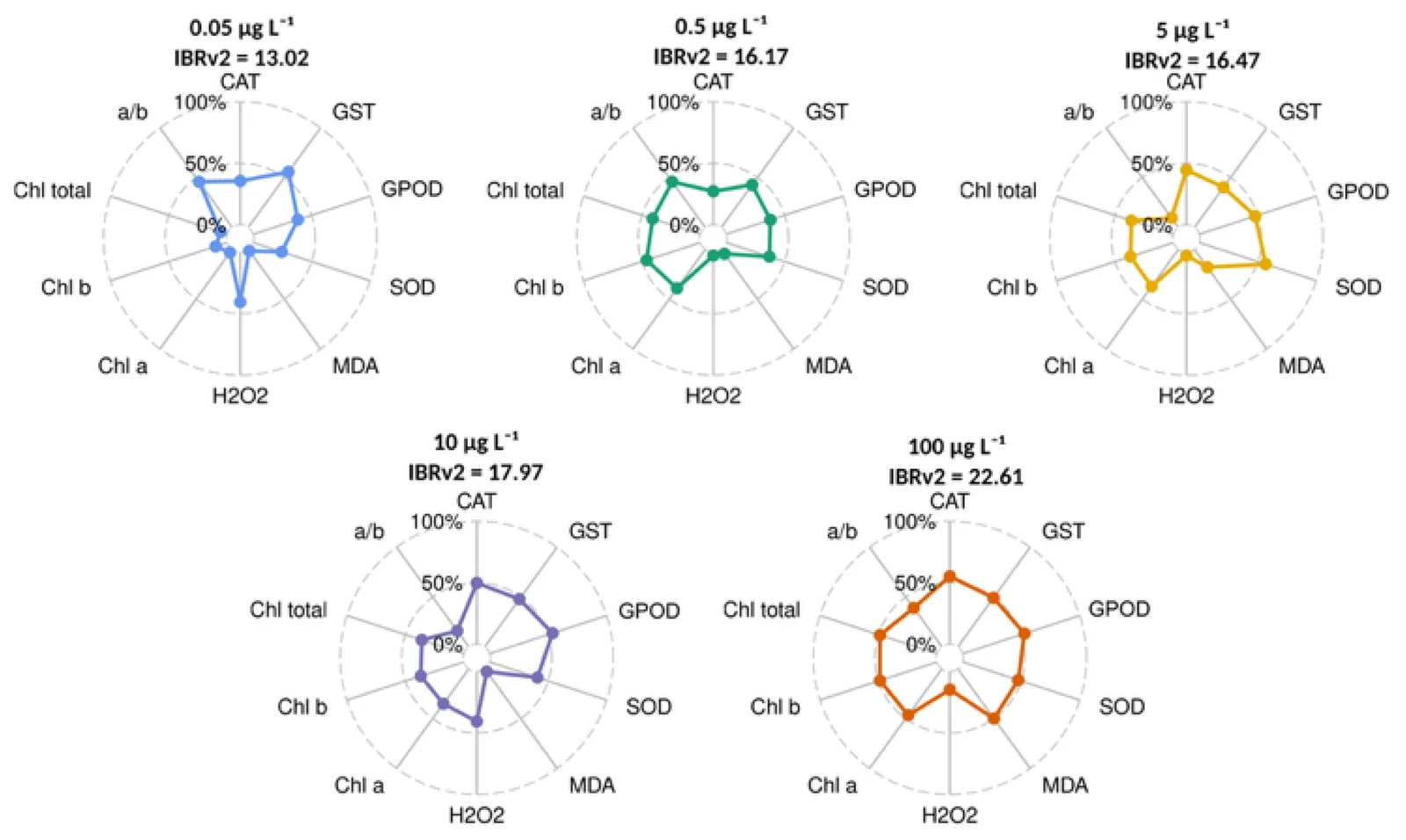
Star plots for the IBRv2 for the shoots and integrated multiple-biomarker response according to each treatment for *B. laevis*.

In the shoots (Figure 4), the IBRv2 star plots revealed concentration-dependent changes in the contribution of individual biomarkers. At 0.05 µg L^−1^, the integrated response was mainly driven by H_2_O_2_ together with GST and GPOD. Between 0.5 and 10 µg L^−1^, antioxidant enzymes remained major contributors, while the contribution of photosynthetic pigments progressively increased. At the highest OTC concentration (100 µg L^−1^), antioxidant enzymes, chlorophyll-related variables, carotenoids, and MDA all contributed substantially to the integrated response, indicating that both oxidative stress and photosynthetic processes were markedly affected. Accordingly, ΣIBRv2 values increased progressively with OTC concentration, reaching the highest value at 100 µg L^−1^.

**Figure 5:**
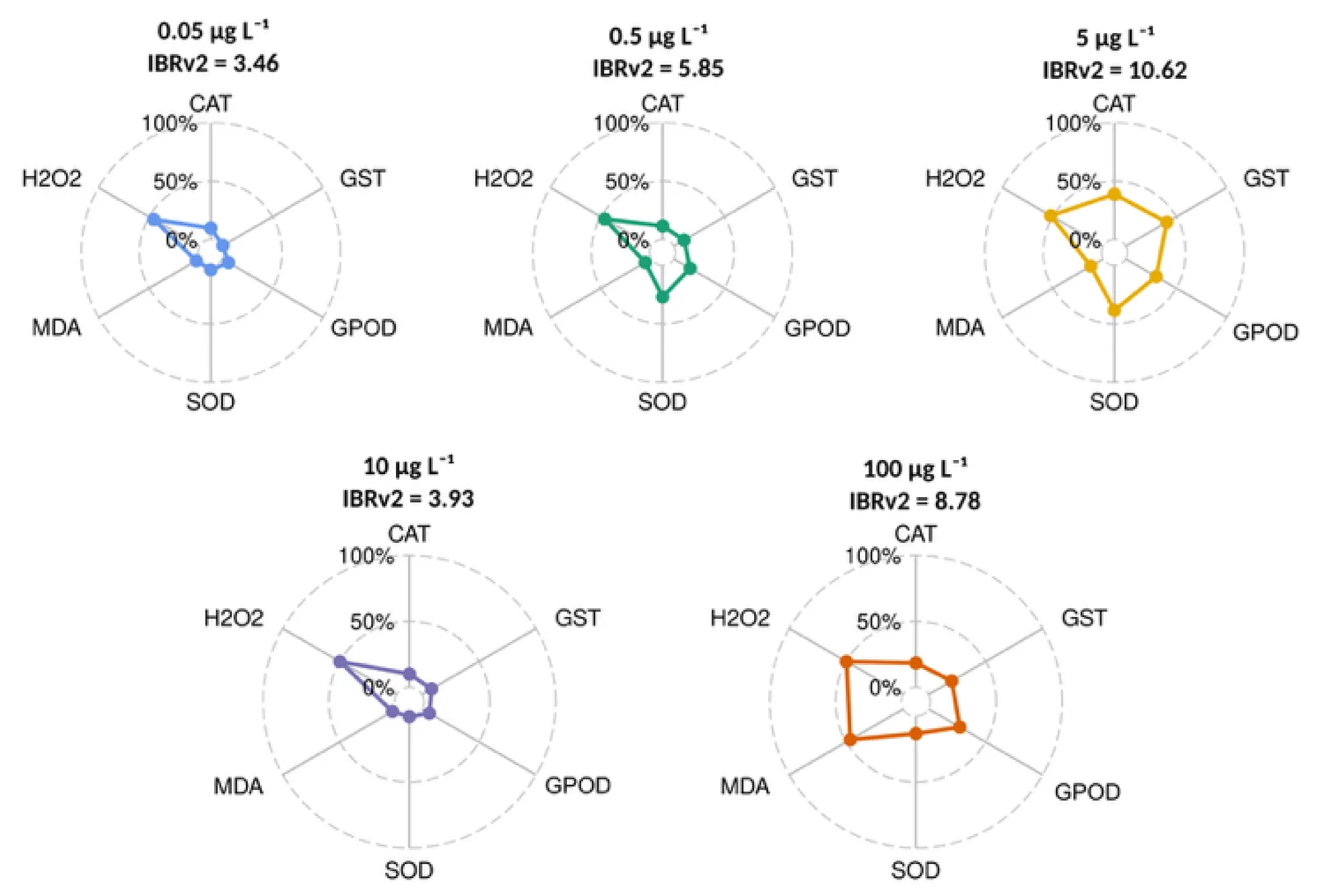
Star plots for the IBRv2 for the roots and integrated multiple-biomarker response according to each treatment for *B. laevis*.

In the roots (Figure 5), H_2_O_2_ was the dominant contributor to the integrated response at 0.05, 0.5, and 10 µg L^−1^, suggesting that hydrogen peroxide accumulation was an early response to OTC exposure. At 5 µg L^−1^, antioxidant enzymes showed a greater contribution while H_2_O_2_ remained an important component of the response. At 100 µg L^−1^, MDA became one of the main contributors together with H_2_O_2_ and antioxidant enzymes, indicating that oxidative stress progressed to membrane lipid peroxidation in the tissue directly exposed to the contaminant. Root ΣIBRv2 values were highest at 5 and 100 µg L^−1^, followed by 0.5, 10, and 0.05 µg L^−1^.

### 2.3. Integrated Analyses: PCA-Biplot and PLS-Biplot

**Figure 6:**
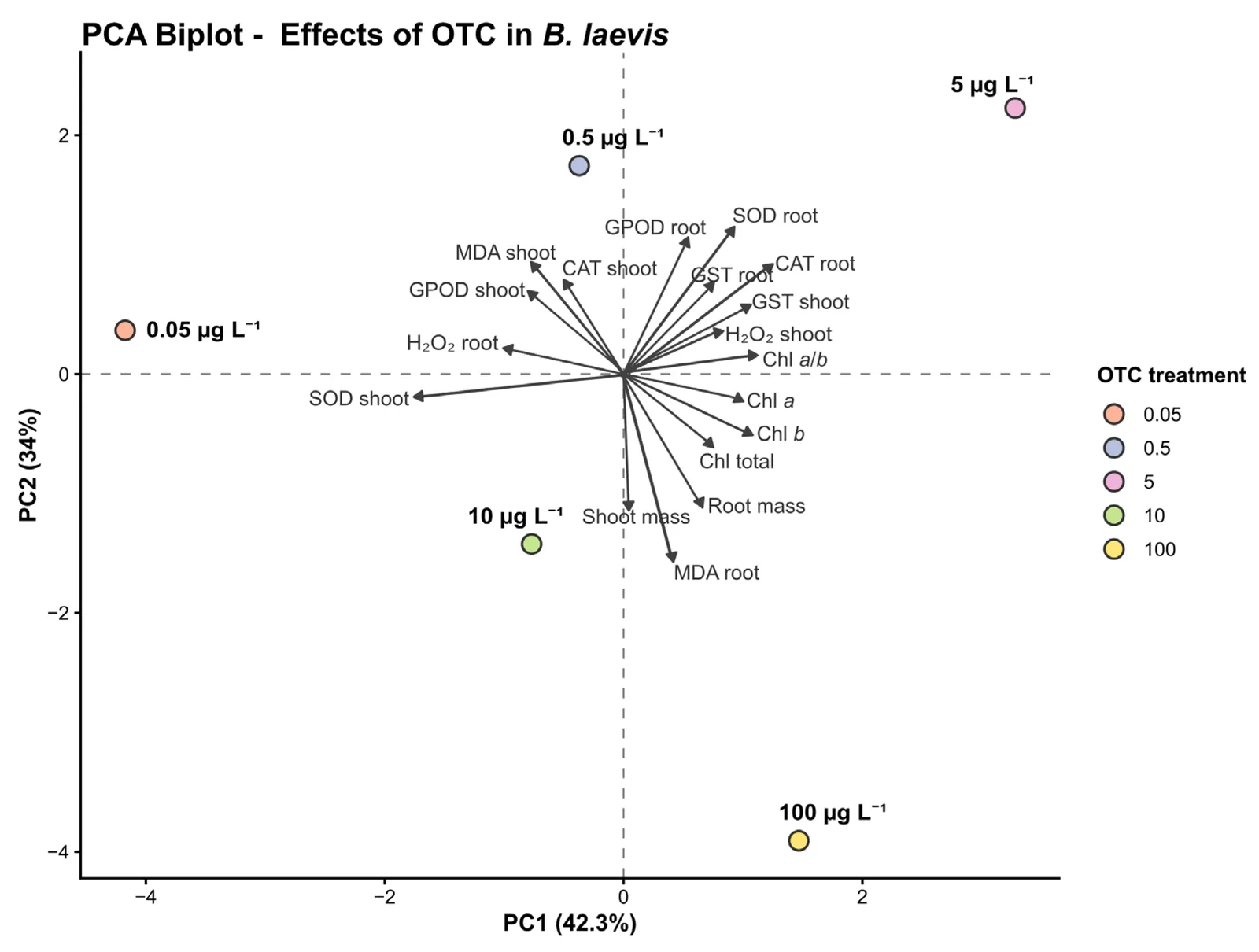
PCA-biplot integrating the variables analyzed according to each treatment of OTC.

The PCA biplot (Figure 6) explained 76.3% of the total variance, with PC1 and PC2 accounting for 42.3% and 34.0%, respectively. Along PC1, shoot antioxidant biomarkers (CAT, GPOD, and SOD), together with root H_2_O_2_, were mainly associated with negative loadings, whereas photosynthetic pigments, root antioxidant biomarkers, and root mass showed positive loadings. PC2 primarily separated root antioxidant biomarkers (CAT, GST, GPOD, and SOD), which showed positive loadings, from OTC mass in shoots and roots, root MDA, and photosynthetic pigments, which were associated with negative loadings.

Treatment distribution in the biplot reflected these response patterns (Figure 6). The 5 µg L^−1^ treatment was positioned in the positive region of both PC1 and PC2 and was associated with root antioxidant responses, particularly CAT, GST, GPOD, and SOD. In contrast, the 100 µg L^−1^ treatment was clearly separated along negative PC2 and was associated mainly with root MDA and OTC accumulation, particularly in roots. The 10 µg L^−1^ treatment was also positioned on the negative side of PC2, closer to the OTC mass variables. The 0.05 µg L^−1^ treatment was strongly separated along negative PC1 and was associated with root H_2_O_2_ and shoot antioxidant responses, whereas the 0.5 µg L^−1^ treatment was positioned toward positive PC2, closer to root antioxidant biomarkers.

**Figure 7:**
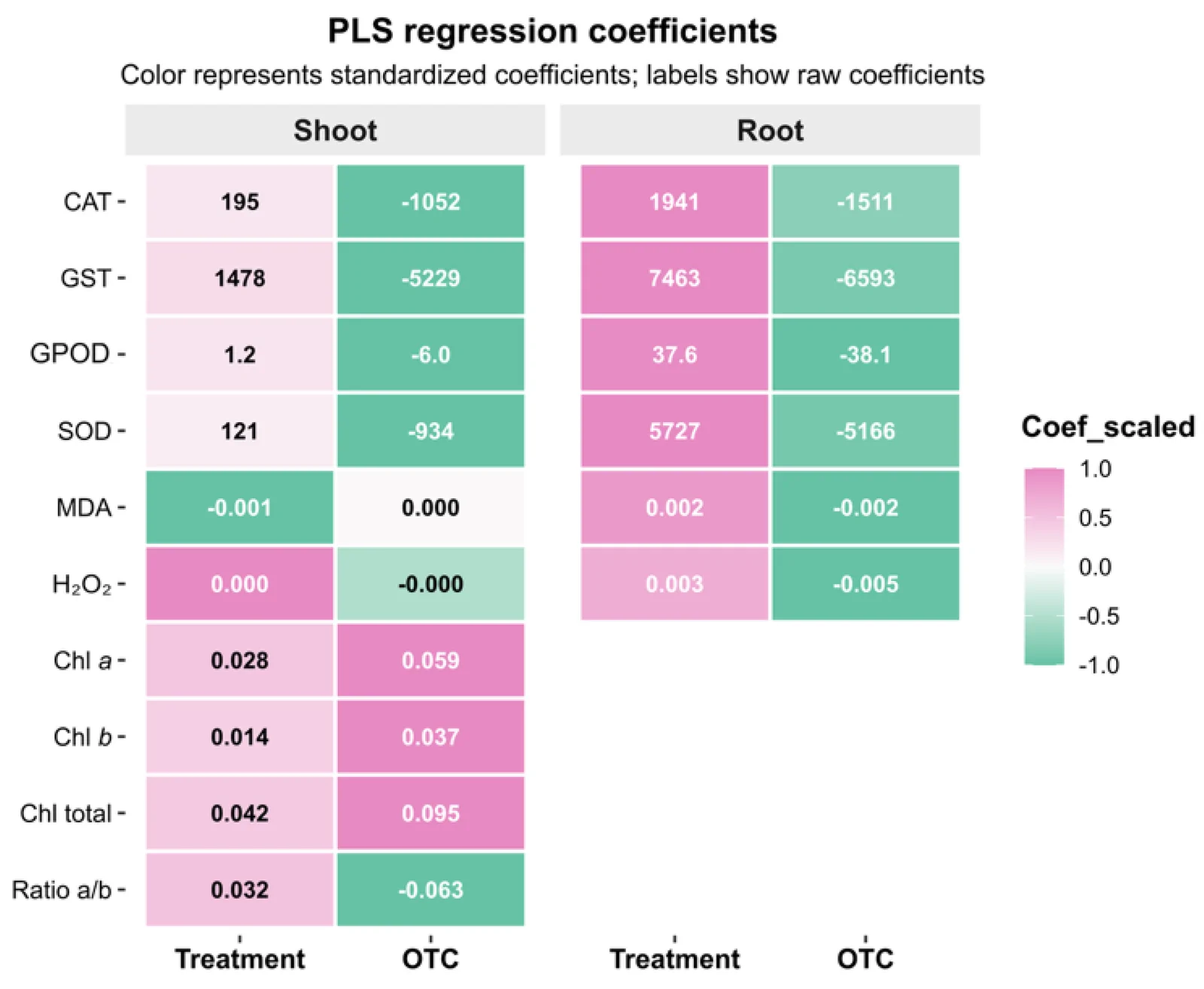
Heatmap of PLS regression coefficients describing the relationships between treatment, OTC accumulation, and biochemical responses in the shoot and root organs of *B. laevis*. Cell colors represent standardized coefficients (−1 to 1), allowing a comparison of the relative direction and strength of predictor effects across variables, whereas numbers correspond to the original regression coefficients.

The first latent component of the PLS explained 82.6% and 85.9% of the variance in the predictor matrix (X) for the shoots and roots, respectively. Overall, the PLS models explained 100% of the predictor variance, while the average variance explained for the response variables (Y) was 29.0% in the shoots and 26.5% in the roots. The PLS (Figure 7; Table S3, Supplementary Materials) revealed a separation between photosynthetic pigments and oxidative stress biomarkers. Photosynthetic variables were closely associated with the exposure gradient, whereas antioxidant responses, particularly in the roots, showed greater dispersion, indicating a stronger contribution to treatment differentiation (Figure 7; Table S3, Supplementary Materials).

In the shoots, the exposure gradient was positively associated with antioxidant biomarkers and photosynthetic pigments, while OTC accumulation exhibited an opposite relationship with most antioxidant responses. In contrast, both exposure and accumulation were positively associated with chlorophyll-related variables. MDA showed a weak positive association with shoot OTC accumulation and a negative association with the exposure gradient (Figure 7; Table S1, Supplementary Materials).

In the roots, antioxidant biomarkers were positively associated with the exposure gradient but negatively related to OTC accumulation. Root OTC mass was located opposite most biochemical responses, indicating contrasting relationships between exposure and accumulation patterns (Figure 7; Table S3, Supplementary Materials). Overall, the PLS analysis showed that treatment concentration and tissue accumulation contributed differently to the observed physiological responses in both organs.

## 3. Discussion

### 3.1. OTC Bioaccumulation, Translocation and Fraction of Initial OTC Accumulated in Plant Tissues in B. laevis Organs

To our knowledge, this is among the first studies to simultaneously assess OTC accumulation and its biochemical and physiological effects in *B. laevis*, a native South American wetland macrophyte with potential for phytoremediation applications. Previous integrative studies evaluating both contaminant uptake and physiological responses to OTC have been limited, and have primarily focused on model species such as *L. minor* [20], which is one of the species that dominates the current ecotoxicological literature on aquatic macrophytes [21]. Consequently, there remains a significant knowledge gap regarding the responses of less-studied species to veterinary pharmaceuticals. This information is particularly relevant for *B. laevis*, a widespread macrophyte in the Pampas region of Argentina, where intensive livestock production coincides with the occurrence of veterinary pharmaceuticals in freshwater environments [27]. Expanding ecotoxicological assessments to native species is therefore essential to improve our understanding of the environmental risks posed by veterinary antibiotics and to identify suitable candidates for nature-based remediation strategies.

The distribution of pharmaceuticals within plants is strongly influenced by their physicochemical properties, particularly hydrophobicity, ionization state, and affinity for plant tissues [14,23]. For example, macrolide antibiotics (anhydroerythromycin A, roxithromycin, clarithromycin and tilmicosin), which generally exhibit higher LogKow values than tetracyclines, tend to accumulate preferentially in roots with limited translocation to aerial organs [28]. Similarly, exposure to high OTC concentrations (>600 µg L^−1^) resulted in greater root than shoot accumulation in *Lythrum salicaria*, *Nymphoides peltata*, *Hydrilla verticillata*, and *Iris wilsonii* [29].

A comparable pattern was observed in *B. laevis*. At low and intermediate concentrations, OTC was distributed relatively evenly between the roots and shoots, whereas root accumulation clearly predominated at 100 µg L^−1^. This shift suggests that translocation is favored at lower concentrations, while root retention becomes increasingly important as external OTC concentrations rise. Macrophytes contribute to antibiotic removal through multiple mechanisms, including root adsorption, uptake, rhizosphere-mediated degradation, and plant metabolism [28,29]. Following root uptake, antibiotics may be transported to aerial tissues via the transpiration stream, where they can be transformed, degraded, or inactivated by plant enzymes and associated cofactors [30].

The relatively high uptake observed at the lowest exposure concentrations may also be linked to the physicochemical properties of OTC. Its hydrophilic nature (LogKow ≈ −0.9) facilitates transport in the aqueous phase and uptake by plant tissues, as previously reported for other hydrophilic veterinary pharmaceuticals such as enrofloxacin [14]. However, as the OTC concentrations increased, accumulation became progressively concentrated in the roots, indicating a shift from translocation to retention. This pattern may result from concentration-dependent partitioning processes, whereby greater OTC availability enhances sorption and sequestration within root tissues, leading to higher root concentrations and lower translocation efficiency. The marked decline in TF values and the predominance of root-associated OTC mass at the highest treatment support this interpretation. Together, these findings might suggest that OTC partitioning within *B. laevis* is governed not only by the physicochemical characteristics of the compound, but also by physiological processes regulating internal transport and storage.

Evidence from other aquatic macrophytes supports the importance of concentration-dependent transport processes. For example, in *E. crassipes* exposed to ciprofloxacin, translocation to aerial tissues was greater at low exposure concentrations (10 µg L^−1^), whereas root accumulation predominated at higher concentrations (50, 100, 500, and 1000 µg L^−1^). This pattern was attributed to changes in plant water relations and reduced transport through the transpiration stream under elevated contaminant levels [31]. Although transpiration was not measured in the present study, a similar mechanism could contribute to the reduced OTC translocation observed at the highest concentration. Future studies should therefore assess transpiration rates and water transport dynamics to better understand OTC migration within *B. laevis*.

Furthermore, the estimated plant-associated OTC recovery fraction indicated that *B. laevis* may have recovered a greater proportion of the initial OTC mass at the lowest exposure concentrations. This finding could suggest that the species may be particularly effective at accumulating environmentally relevant OTC concentrations from the aqueous phase. However, because residual OTC concentrations were not measured at the end of the experiment, these values should be interpreted as the fraction of OTC recovered in plant biomass rather than as a direct estimate of removal efficiency.

The saturation in roots at the highest exposure treatment of OTC and lack of translocation could be explained by the properties of the compound, since OTC is a multi-ionizable compound whose speciation depends on the pH of the medium [32]. At pH values between approximately 4–8, including those of the Hoagland solution used in the present study, the molecule occurs predominantly in its zwitterionic form (pKa ≈ 7.3) [33]. In this state, OTC simultaneously carries positive and negative charges, which can modify its capacity to permeate the membranes, interactions with dissolved ions, and ultimately, its bioavailability for root uptake.

In rice (*Oryza sativa*), Boonsaner and Hawker [33] reported a rapid accumulation of OTC in roots, reaching concentrations of up to 800 mg kg^−1^ within the first 24 h of exposure. Although the experiment continued for 15 days, root concentrations reached a plateau during the first days of exposure, suggesting that OTC accumulation may be constrained by the uptake capacity of root tissues and/or intracellular detoxification processes, including glutathione-mediated transformation. Moreover, the authors demonstrated that tetracycline uptake and root bioaccumulation were better explained by LogDow (−2.30) than by LogKow, emphasizing that the pH-dependent hydrophobicity of ionizable compounds is a more appropriate predictor of their behavior in plants [32]. In the present study, the initial pH of the exposure medium ranged between 6 and 7, conditions under which OTC is expected to occur predominantly in its zwitterionic form. However, pH was not measured at the end of the 48-h exposure period, preventing us from determining whether changes in the exposure medium may have altered OTC speciation, and consequently, its bioavailability and uptake over time. This represents a limitation when interpreting the mechanisms underlying OTC accumulation. Therefore, the saturation in roots at the highest OTC concentration observed in the present study could reflect a limitation in uptake and/or intracellular processing; however, this interpretation should be considered with caution. This hypothesis remains speculative because neither changes in pH and OTC speciation during exposure nor the mechanisms involved in OTC transport and intracellular processing were directly assessed, and the exposure period (48 h) was considerably shorter than that used by Boonsaner and Hawker [33].

Regarding the potential valorization of *B. laevis* biomass containing accumulated OTC, studies addressing the management and valorization of plant biomass contaminated with antibiotics remain scarce, particularly for OTC. Recently, Wazeri et al. [34] employed an experimental wetland containing *Azolla* sp. to treat tetracycline-contaminated water over a 300-day period. After its use in the treatment system, the exhausted plant biomass was not simply discarded but was subjected to thermal conversion to produce biochar, thereby providing a potential valorization pathway for the residual biomass rather than generating an additional waste stream. The authors also evaluated the economic feasibility of the integrated system and reported that biochar valorization, together with the potential benefits associated with carbon sequestration, could contribute to its economic viability. In addition, thermal conversion contributed to the stabilization of low-toxicity transformation products [34]. A similar strategy could potentially be explored for *B. laevis*, particularly given its relatively high biomass production compared with small floating macrophytes such as *Azolla*. However, the fate of accumulated OTC and its transformation products during thermal treatment would need to be specifically assessed before biochar production from OTC-containing *B. laevis* biomass could be considered a safe and viable strategy. Such an approach could potentially integrate contaminant management with biomass valorization within a circular-economy framework.

### 3.2. Physiological and Oxidative Stress Biomarkers in B. laevis: A Multiple-Biomarker Approach

Exposure to OTC has produced contrasting responses in antioxidant and detoxification systems among aquatic macrophytes. In *T. barbieri*, GST and glutathione reductase (GR) activities decreased under some OTC treatments (25 µg L^−1^) compared with the controls [19]. The authors suggested that GST-mediated biotransformation played a limited role in OTC detoxification during the 24 h exposure period. However, GST fulfills multiple physiological functions, including both xenobiotic detoxification and protection against oxidative stress, making its response difficult to interpret solely in terms of biotransformation capacity.

A similar inhibitory pattern was observed in *V. natans*, where CAT and GPOD activities decreased following exposure to OTC concentrations higher (100 mg L^−1^ for 14 days) than those tested in our study [35], and for CAT in *E. densa* at the highest concentration tested (25 µg L^−1^ for 24 h) [19]. In our study, shoot CAT, GPOD, and SOD activities also tended to decline with increasing OTC concentrations. Because these enzymes constitute the main antioxidant defenses involved in the detoxification of ROS, their inhibition may indicate either a reduced requirement for antioxidant activity or a suppression of enzymatic defenses under OTC exposure. The concomitant decrease in MDA and increase in GST activity at the highest treatment suggest that oxidative damage could be controlled by GST, since GST can conjugate reduced glutathione (GSH) to membrane lipid peroxides, detoxifying such products directly [36,37]. Thus, antioxidant responses to OTC may vary depending on exposure concentration, duration, and species-specific physiological traits of the plants [35]. Previous studies with *B. laevis* exposed to the ionophore antibiotic monensin did not detect significant changes in antioxidant biomarkers in either the roots or shoots [26]. These contrasting responses may reflect differences in the uptake, accumulation, and translocation patterns of the two pharmaceuticals, which belong to distinct classes of veterinary antibiotics and differ markedly in their physicochemical properties (e.g., lipophilicity) and mechanisms of action.

In contrast to antioxidant biomarkers, photosynthetic pigments increased with OTC exposure. This response differs from that reported for *V. natans*, where chlorophyll concentrations remained unchanged following OTC exposure [35], and from previous experiments in *B. laevis* exposed to monensin, which also showed no significant effects on pigment content [26]. Similar stimulatory responses have been described for other pharmaceuticals, supporting the concept of hormesis, whereby low or moderate contaminant concentrations induce compensatory or adaptive physiological responses. For example, ofloxacin concentrations ranging from 1 to 1000 μg L^−1^ stimulated both cell density and pigment production in *Chlorella pyrenoidosa* after 21 days of exposure [38]. Likewise, increases in chlorophyll content under chemical stress have been associated with protective mechanisms that limit pigment degradation and preserve photosynthetic structures [39].

The relationship between oxidative stress biomarkers and photosynthetic pigments was further supported by the IBRv2 analysis. The highest OTC treatment exhibited the greatest ΣIBRv2 value, with photosynthetic pigments and antioxidant biomarkers representing the main contributors to the integrated response. These results indicate that pigment alterations, together with the activation of antioxidant defenses, were the primary physiological processes underlying the stress response to OTC exposure.

The IBRv2 proved to be a valuable tool for integrating multiple biochemical and physiological endpoints into a single index, thereby providing a comprehensive assessment of the overall biological response to emerging contaminants such as antibiotics [40,41]. Nevertheless, some limitations should be considered when interpreting the index. Because IBRv2 depends on the selection of biomarkers, the omission of relevant endpoints may lead to an incomplete characterization of the organism’s physiological status. In addition, the normalization procedure used to calculate the index can be influenced by outliers and experimental variability, potentially affecting the relative contribution of individual biomarkers [41]. Despite these limitations, the consistent increase in ΣIBRv2 with OTC concentration supports the sensitivity of this integrative approach for detecting and summarizing sublethal biochemical responses in *B. laevis*.

Nevertheless, the ecological significance of the increase in photosynthetic pigments observed in the present study remains uncertain. Although chlorophyll concentrations increased following OTC exposure, higher pigment levels do not necessarily reflect enhanced photosynthetic performance, as no direct measurements of photosynthetic activity (e.g., chlorophyll fluorescence or gas exchange) were performed. Previous studies have shown that low levels of environmental stress commonly stimulate chlorophyll accumulation, suggesting that chlorophylls play a central role in plant stress biology by contributing to the maintenance of physiological functioning under adverse conditions [42]. Within this framework, the increase in chlorophyll content observed here may represent an adaptive or hormetic response to OTC exposure rather than an improvement in photosynthetic efficiency. According to the hormesis studies, stimulation at low stress levels may help maintain physiological homeostasis, preventing chlorophyll concentrations from declining below control levels as stress intensity increases [42]. Therefore, the increase in pigment content should be interpreted as a physiological adjustment to OTC exposure, whereas its functional consequences for plant performance remain unclear and warrant further investigation.

In the roots, antioxidant biomarkers remained largely unchanged across OTC treatments, a pattern consistent with previous observations in *B. laevis* exposed to monensin [26]. The only exception was H_2_O_2_, which decreased at 5 µg L^−1^ but returned to values comparable to the control at the highest exposure concentration. Although individual biomarkers did not show significant treatment-related effects, the IBRv2 analysis revealed a clear trend. H_2_O_2_ was the main contributor to the integrated response across all treatments, whereas MDA became increasingly important at the highest concentration, resulting in the greatest ΣIBRv2 value. These results suggest that subtle physiological adjustments occurred in roots that were not fully captured by the univariate analyses.

This pattern contrasts with the more pronounced biochemical responses observed in shoots and is consistent with the differential partitioning of OTC between plant organs. At low exposure concentrations, OTC was more efficiently translocated to aerial tissues, whereas root retention predominated at the highest concentration. Despite accumulating most of the OTC at 100 µg L^−1^, roots showed neither marked activation of antioxidant defenses nor clear evidence of oxidative damage. Although H_2_O_2_ and MDA contributed to the IBRv2 profile at the highest treatment, suggesting the onset of oxidative processes, these changes were insufficient to elicit significant alterations in the antioxidant defense system. Similarly, Li et al. [43] reported no significant biochemical changes in the roots of Triticum aestivum exposed to OTC at concentrations up to 5 mg L^−1^, with significant responses occurring only at concentrations above 10 mg L^−1^, far exceeding the environmentally relevant levels. Collectively, these findings indicate that root tissues can accumulate and retain substantial amounts of OTC while maintaining physiological homeostasis, suggesting a relatively high tolerance to short-term exposure.

From an applied perspective, this combination of high accumulation capacity and limited biochemical disturbance highlights the potential role of *B. laevis* roots in the sequestration of veterinary antibiotics in freshwater environments. Nevertheless, longer exposure periods and the evaluation of additional physiological and metabolic endpoints are needed to determine whether this apparent tolerance can be sustained under chronic exposure conditions.

### 3.3. Integrative Statistical Approaches to Assess the Ecotoxicological Effects of OTC in B. laevis

The multivariate analyses provided complementary insights into the responses of *B. laevis* to OTC exposure. The PCA indicated a shift from antioxidant responses at low exposure concentrations toward OTC accumulation, photosynthetic pigments, and oxidative damage at higher concentrations. In particular, the 5 µg L^−1^ treatment was associated with root antioxidant defenses, whereas the 10–100 µg L^−1^ treatments were more closely related to OTC accumulation and root MDA. The PLS analysis further suggested that exposure concentration and tissue accumulation exerted different effects on plant responses. While the exposure gradient was generally associated with the activation of antioxidant and physiological responses, OTC accumulation showed an opposite relationship with several biomarkers. This pattern suggests that exposure may trigger stress responses, whereas excessive accumulation could attenuate or inhibit some defense mechanisms.

Although both analyses identified photosynthetic pigments as important response variables, they addressed different aspects of the dataset. PCA provided an overview of treatment-associated response patterns, whereas PLS allowed the separation of exposure- and accumulation-related effects. The combined use of both approaches therefore improved the interpretation of the complex physiological responses induced by OTC in *B. laevis*.

PLS has been widely applied in ecological studies because it combines the strengths of principal component analysis and multiple regression and is particularly useful when predictor variables are highly correlated [44]. Applications include the identification of environmental drivers of phytoplankton dynamics and the assessment of metabolic responses of marine mussels exposed to wastewater treatment plant effluents [45]. However, to our knowledge, its use remains uncommon in ecotoxicological bioassays integrating contaminant accumulation, oxidative stress biomarkers, and physiological responses in animals [46] or even in aquatic plants. Therefore, the combined use of PCA and PLS may provide a useful framework for disentangling exposure- and accumulation-related effects in phytotoxicological studies.

## 4. Materials and Methods

### 4.1. Chemicals and Physico-Chemical Properties of OTC

Oxytetracycline (OTC; 5-hydroxytetracycline hydrochloride, C_22_H_24_N_2_O_9_; CAS No. 2058-46-0) was purchased from Sigma-Aldrich (Buenos Aires, Argentina) as an HPLC-grade standard with high purity (≤3.5% total related substances). OTC has a molecular weight of 460.43 g mol^−1^, a density of 1.634 g cm^−3^ at 20 °C, and a water solubility of 313 mg L^−1^ at 25 °C. It is an amphoteric compound with reported pKa values of 3.27 and 9.50, corresponding to the ionization of acidic and tertiary amine functional groups, respectively. The compound exhibits a melting point of 184.5 °C and a LogKow of −0.90, indicating hydrophilicity (PubChem, https://pubchem.ncbi.nlm.nih.gov).

A concentrated stock solution of OTC (1 mg mL^−1^) was prepared in HPLC analytical-quality ethanol (96%),to ensure proper dilution. Working solutions were obtained by serial dilution of the stock solution and used to prepare the experimental nominal concentrations of 0.05, 0.5, 5, 10, and 100 μg L^−1^. This concentration range was selected to encompass the environmentally relevant OTC levels reported in aquatic ecosystems (Table S2, Supplementary Materials).

### 4.2. Seeds Collection and Seedlings Growth

Seeds of *B. laevis* were collected from natural populations at Arroyo Tajamar (37°52′54″ S, 57°58′11.8″ W) in Argentina during the early autumn of 2023 (Southern Hemisphere), following the flowering period (Figure S1, Supplementary Materials). Seeds were surface-sterilized with 10% commercial bleach for 10 min and subsequently rinsed several times with distilled water. To promote germination, seeds were mechanically scarified under a stereomicroscope (4× magnification) following the procedure described by Truchet et al. [26]. After germination, seedlings were cultivated individually in pots containing Argiudoll soil, selected for its similarity to the species’ natural substrate. Plants were maintained in a growth chamber at 24 °C under an illumination of 8000 lux. After two months of growth, uniform seedlings were selected and transferred to glass vessels containing dicotyledon Hoagland nutrient solution (pH: 6–7; Table S3, Supplementary Materials). A total of 36 two-month-old seedlings (*n* = 6 per treatment) were randomly assigned to six experimental treatments and exposed for 48 h. During the exposure period, individual seedlings were maintained in 330 mL glass flasks covered to minimize light penetration and kept under the same temperature and photoperiod conditions used during cultivation (Figure S2, Supplementary Materials).

### 4.3. Experimental Design and Organ Plant Collection

Prior to exposure, baseline morphological parameters, including total plant length (50.08 ± 8.23 cm), root length (23.91 ± 7.54 cm), and shoot length (26.17 ± 2.49 cm), were measured using a digital caliper and expressed in centimeters (cm). Total fresh biomass (8.96 ± 2.25 g) was determined with a granatary balance (OHAUS^®^ Pioner PX, Buenos Aires, Argentina).

Experimental concentrations consisted of 0 (solvent control), 0.05, 0.5, 5, 10, and 100 µg L^−1^ OTC. The solvent control contained the same concentration of ethanol (99.5%, Ciccarelli, Buenos Aires, Argentina) used as the carrier in the highest OTC treatment −0.1% (*v*/*v*). Seedlings were exposed for 48 h in Hoagland solution under controlled temperature (24 ± 1 °C) and a photoperiod representative of austral spring conditions (Figure S2, Supplementary Materials).

At the end of the exposure period, morphological parameters were measured (total plant length: 52.44 ± 7.62 cm; root length: 22.93 ± 7.62 cm; shoot length: 29.51 ± 2.63 cm and total fresh biomass: 9.81 ± 2.43 g) to calculate the mass of OTC in plants (Supplementary Materials, Table S4). A leaf subsample (0.2 g fw) was collected for photosynthetic pigment determination. The remaining plant material was separated into roots and shoots (leaves + stem). For each organ, 0.3 g was reserved for MDA analysis, 1 g for the determination of oxidative stress biomarkers, and the remaining tissue for OTC bioaccumulation assessment (Figure S2, Supplementary Materials). All samples were immediately frozen in liquid nitrogen and stored at −80 °C until analysis.

### 4.4. OTC Analytical Determination

#### 4.4.1. Hoagland Exposure Solution

Aliquots (0.9 mL) of the exposure solutions were filtered using 0.22 μm nylon syringe filters and collected in 1.5 mL amber glass vials. Afterward, 0.1 mL of HPLC-grade methanol was added prior to instrumental analysis by ultra-performance liquid chromatography coupled to tandem mass spectrometry (UPLC-MS/MS; Waters Xevo TQ-S Micro™, Buenos Aires, Argentina).

External calibration was performed with OTC standards prepared in OTC-free ultrapure water (90:10, *v*/*v*) covering a concentration range between 0.1 and 200 μg L^−1^. Exposure media samples were concentrated tenfold before analysis, allowing a method LOD and LOQ of 0.01 and 0.03 μg L^−1^, respectively.

Measured OTC concentrations (mean ± SD) were 0.02 ± 0.01, 0.47 ± 0.03, 4.75 ± 0.21, 9.92 ± 0.04, and 104.5 ± 2.12 μg L^−1^, corresponding to nominal concentrations of 0.05, 0.5, 5, 10, and 100 μg L^−1^, respectively. Additional details of the chromatographic and mass spectrometric analyses are described below.

#### 4.4.2. Plant Material

Extraction and determination of OTC in plant tissues were based on the procedure reported by Chen et al. [47] for wetland macrophytes exposed to antibiotics in hydroponic systems, with adaptations for *B. laevis*. Frozen tissues were first pulverized under liquid nitrogen to obtain a homogeneous powder and minimize analyte degradation. Subsequently, 0.2 g of homogenized tissue were weighed into 50 mL polypropylene centrifuge tubes and mixed with 2 mL of citrate buffer (0.09 M citric acid and 0.01 M sodium citrate, pH 3.0). After manual shaking and vortex mixing for 1 min, 5 mL of HPLC-grade acetonitrile containing 1% acetic acid was added. Samples were extracted by orbital shaking at 250 rpm for 30 min and subjected to two consecutive 15 min ultrasonic treatments.

For the dispersive clean-up step, a salt mixture consisting of 1 g anhydrous MgSO_4_, 0.25 g NaCl, 0.25 g C_6_H_8_O_7_, and 0.25 g Na_3_C_6_H_5_O_7_ was added, followed by vigorous shaking for 1 min. Tubes were then centrifuged at 3500 rpm for 15 min. An aliquot (1 mL) of the organic phase was transferred to a 2 mL tube containing 50 mg PSA and 150 mg anhydrous MgSO_4_ for QuEChERS clean-up. After vortexing for 1 min and centrifugation at 4000 rpm for 10 min, 600 μL of the extract was combined with 400 μL of ultrapure water supplemented with 0.5 mM EDTA. The resulting solution was filtered through a 0.22 μm nylon membrane, transferred to amber vials, and stored at 4 °C until UPLC-MS/MS analysis.

Instrumental determination of OTC was carried out using an Acquity UPLC I-Class system coupled to a XEVO TQ-XS triple quadrupole mass spectrometer (Waters, Milford, MA, USA) operating in positive electrospray ionization mode (ESI+). Chromatographic separation was achieved on an Acquity UPLC HSS C18 column (100 × 2.1 mm, 1.8 μm particle size) maintained at 45 °C. The flow rate was set at 0.3 mL min^−1^, the injection volume at 10 μL, and the total run time at 6 min. Mobile phase A consisted of water (95:5, *v*/*v*), whereas mobile phase B was methanol. Both phases contained 0.1 mM ammonium acetate and 0.01% formic acid. The gradient program increased phase B from 10% to 100% by 3 min, maintained this composition until 5 min, and returned to 10% at 6 min. Mass spectrometric parameters were optimized as follows: capillary voltage 3 kV; source temperature 150 °C; desolvation temperature 500 °C; cone gas flow 150 L h^−1^; desolvation gas flow 800 L h^−1^ (nitrogen); and collision gas flow 0.15 mL min^−1^ (argon, 99.995%). Data processing was performed with MassLynx v4.2 software.

Quantification was conducted in multiple reaction monitoring (MRM) mode. OTC identification was based on a retention time of 2.2 min and the detection of two characteristic product ions derived from the precursor ion. The transition Q: 461 → 444 was selected for quantification, whereas transition q: 461 → 426 was used for confirmation. The MRM mode operated with a cone voltage of 30 V and collision energies for Q and q of 15 eV. Positive identification was confirmed when the Q/q ion ratio of the sample matched that of the reference standard within ±20%. Calibration curves ranging from 0.01 to 200 μg L^−1^ were prepared in OTC-free ultrapure water (90:10, *v*/*v*), yielding coefficients of determination (R^2^) greater than 0.99.

#### 4.4.3. Quality Controls

Each analytical sequence included solvent blanks, fortified solvent samples, and fortified plant tissue samples analyzed in triplicate. Recovery experiments were performed using OTC-spiked root and shoot tissues processed through the complete extraction protocol. Solvent blanks were periodically analyzed to verify the absence of contamination and carryover effects, while continuing calibration verification standards were injected after every five samples.

The LOD was established as the minimum concentration producing a signal-to-noise ratio (S/N) ≥ 3. The LOQ corresponded to the lowest matrix fortification level meeting the following criteria simultaneously: recovery between 70 and 120% of the spiked concentration, relative standard deviation (RSD) ≤ 15%, and S/N ≥ 10. Method accuracy and precision were assessed through recovery assays using OTC-fortified plant tissues. For this purpose, 0.2 g aliquots of control root and shoot tissues were spiked with 10 μL of an OTC working solution (100 μg mL^−1^), resulting in a fortification level of 500 μg kg^−1^ fw, and subsequently subjected to the complete extraction and clean-up procedure. Average recoveries were 112.2 ± 25.8% for the roots and 122.8 ± 13.2% for the shoots, corresponding to RSD values of 23.0% and 10.7%, respectively. Thus, the recovery obtained for shoots slightly exceeded the predefined upper acceptance limit of 120%, whereas the RSD obtained for roots exceeded the predefined criterion of ≤15%. The LOD values were 2.02 and 1.78 μg kg^−1^ fw for the roots and shoots, respectively, whereas the LOQ was 2.50 μg kg^−1^ fw for both tissues. Matrix effects were <20%.

### 4.5. Biochemical and Physiological Biomarkers

Antioxidant enzyme activities were determined in the roots and shoots following the procedures described by Pflugmacher [48]. Briefly, approximately 1 g of fresh tissue was frozen in liquid nitrogen and ground with a mortar and pestle to obtain a fine powder. The powder was transferred to a 25 mL glass beaker containing 2 mL of extraction buffer composed of 0.1 M Na_3_PO_4_ buffer (pH 6.5), 1.4 mM dithiothreitol, 1 mM EDTA, and 5 mM phenylmethylsulfonyl fluoride to prevent protein degradation. Samples were mixed on a magnetic stirrer for 10 min under cold conditions. The homogenates were then transferred to 2 mL tubes and centrifuged at 10,000× *g* for 10 min at 4 °C. Protein extracts were used for the assessment of CAT, GST, GPOD, and SOD activities, as well as H_2_O_2_ content.

Enzyme activities were expressed as nanokat mg^−1^ protein min^−1^. CAT, GST, GPOD, and SOD activities were measured according to the methods of Claiborne [49], Habig et al. [50], Pütter [51], and Scebba et al. [52], respectively, and protein content was assessed through the Bradford [53] method.

Contents of H_2_O_2_ were quantified following Bellincampi et al. [54] and expressed as nmol H_2_O_2_ mg^−1^ fw. Lipid peroxidation was evaluated by measuring MDA levels in approximately 0.3 g of fresh tissue according to Shi et al. [55], and results were expressed as nmol MDA g^−1^ fw.

Photosynthetic pigments, including chlorophyll *a* (Chl *a*), chlorophyll *b* (Chl *b*), total chlorophyll, and the Chl *a*/Chl *b* ratio, were extracted and quantified following the method of Inskeep and Bloom [56]. All biochemical and physiological endpoints were determined spectrophotometrically in triplicate using a SPECTROstar Nano microplate reader (BMG LABTECH, Buenos Aires, Argentina).

### 4.6. Data Analyses

The mass of OTC accumulated in the roots and shoots was calculated by multiplying the OTC concentration measured in each organ (µg kg^−1^ fw) by its corresponding fresh biomass (g) at the end of the experience. Total OTC mass per plant was estimated as the sum of the masses accumulated in the roots and shoots. The relative distribution of OTC within the plant was expressed as the percentage contribution of each organ to the total accumulated mass. The distribution (%) was calculated as the OTC mass in a given organ divided by the total OTC mass accumulated in the plant, multiplied by 100 (Equation (1)):

(1) $$Distribution\;(\%)=\frac{{Mass}_{organ}}{{Mass}_{total}}\times 100$$

To estimate the contribution of plant uptake to OTC dissipation (Equation (2)), the percentage of the initial OTC mass recovered in plant tissues was calculated as the ratio between the mass accumulated in the roots, shoots, or whole plants and the initial OTC mass present in the exposure medium. The initial OTC mass in the exposure medium was calculated using the measured concentration in water and the volume of each exposure vessel (330 mL). Subsequently, the proportion of OTC recovered in the roots, shoots, and whole plants was estimated by relating the mean accumulated mass in each organ (or total plant) to the mean initial OTC mass in water for each treatment. This was calculated based on a mass-balance approach commonly used in phytoremediation studies [57,58]. As residual OTC concentrations were not determined at the end of the experiment, these values represent the fraction of the initial OTC mass accumulated in plant tissues rather than the actual removal efficiency of the system.

(2) $$Fraction\;of\;initial\;OTC\;accumulated\;in\;plant\;tissues\;(\%)=\frac{{Mass}_{plant}\;}{C_{0}\times V}\times 100$$

where *C*_0_ is the concentration of OTC measured at the beginning of the exposure (µg L^−1^), and *V* is the volume of the exposure media (0.33 L). The mass represents the total mass of the plant measured as the concentration of OTC in the plant (µg g^−1^) and biomass of the organ (g).

The bioaccumulation factor (BAF) (Equation (3)) was calculated for the roots and shoots as the ratio between the OTC concentration measured in plant tissues (*C_organ_*, µg kg^−1^ fw) and the corresponding measured OTC concentration in the exposure medium (*C_water_*, µg L^−1^). BAF values (L kg^−1^) were used to evaluate the accumulation capacity of each organ. A BAF value > 1 indicates preferential bioaccumulation to the shoots or roots from the solution, whereas values < 1 indicate no bioaccumulation in the organs.

(3) $${BAF}_{organ}=\frac{C_{organ}}{C_{water}}$$

The translocation factor (TF) was calculated as the ratio between OTC concentrations in the shoots (*C_shoot_*) and roots (*C_root_*), providing an estimate of the efficiency of OTC translocation from belowground to aboveground tissues (Equation (4)). A TF value > 1 indicates preferential translocation to shoots, whereas values < 1 indicate retention of the compound in roots.

(4) $$TF=\frac{C_{shoot}}{C_{root}}$$

Data obtained for OTC bioaccumulation, oxidative stress biomarkers, and photosynthetic pigments were tested for normality using the Shapiro–Wilk test and for homogeneity of variances using Bartlett’s test. When necessary, data were log-transformed prior to statistical analyses. Differences among treatments were evaluated separately for roots and shoots using one-way analysis of variance (ANOVA), with treatment as the fixed factor. When significant differences were detected, Tukey’s honestly significant difference (HSD) test was used for post hoc comparisons. For OTC concentrations and mass in plants, pairwise comparisons were performed using the Wilcoxon rank-sum test.

The Integrated Biological Response index version 2 (IBRv2) was calculated according to Sánchez et al. [40], using the control treatment as the reference condition. Biomarker responses were log-transformed, standardized, and integrated into a single index by summing the absolute deviations from the reference values. Higher IBRv2 scores indicate greater biochemical and physiological disturbance. Six biomarkers were included in the root IBRv2 calculation (CAT, GST, GPOD, H_2_O_2_, MDA, and SOD), whereas ten biomarkers were considered for the shoots (CAT, GST, GPOD, H_2_O_2_, MDA, SOD, Chl *a*, Chl *b*, total chlorophylls and Chl *a*/Chl *b* ratio), reflecting organ-specific biochemical and physiological functions. Standardized biomarker responses were visualized using radar plots to illustrate the relative contribution of each biomarker to the overall integrated response.

To integrate the biological and accumulation data, principal component analysis (PCA) and partial least squares regression (PLS) were performed. PCA biplots were constructed using all measured biomarkers together with OTC mass accumulated in the roots and shoots to explore patterns of variation among treatments and identify associations between physiological responses and OTC accumulation in *B. laevis.* PLS analysis was subsequently applied to identify the variables most strongly associated with the OTC exposure gradient and to evaluate the relative contribution of each biomarker to treatment differentiation and OTC mass for each organ. All statistical analyses and graphical representations were performed in RStudio (version 4.5.2; [59]) using the *packages tidyverse*, *dplyr*, *ggplot2*, *ggradar*, *stringr*, *FactoMineR*, *factoextra*, and *pls*.

## 5. Conclusions and Future Directions

This study shows that *B. laevis* is capable of taking up, bioaccumulating, and translocating OTC at environmentally relevant concentrations during short-term exposure. BAF values exceeded 1 in all treatments, confirming OTC accumulation in plant tissues relative to its concentration in the exposure medium. OTC partitioning varied with increasing exposure concentration, shifting from predominant accumulation in shoots at low concentrations to greater retention in roots at the highest treatment. This pattern suggests concentration-dependent differences in OTC uptake and translocation; however, the physiological mechanisms underlying this distribution were not directly assessed.

Despite OTC accumulation, biochemical responses were relatively moderate. Antioxidant biomarkers in shoots tended to decrease with increasing exposure, whereas roots exhibited limited alterations. In contrast, photosynthetic pigments increased with OTC concentration. However, because photosynthetic activity was not directly assessed, for example, through chlorophyll fluorescence or gas exchange measurements, increased pigment concentrations cannot be interpreted as evidence of enhanced photosynthetic performance. The combined use of IBRv2, PCA, and PLS analyses revealed distinct relationships between OTC exposure, tissue accumulation, and plant physiological responses. While exposure concentration was associated with changes in several biological endpoints, tissue accumulation showed different relationships with these responses, indicating that greater OTC accumulation was not necessarily accompanied by greater biochemical disturbance under the conditions tested.

Overall, *B. laevis* showed the capacity to accumulate OTC while maintaining relatively stable biochemical responses during the 48-h exposure period. These findings support its potential for further investigation in phytoremediation-oriented studies in Pampean freshwater ecosystems affected by livestock activities, but they should not be interpreted as evidence of phytoremediation efficiency. In particular, residual OTC concentrations in the exposure medium were not measured after exposure; therefore, the actual OTC removal from water could not be quantified, and plant-associated OTC recovery represents bioaccumulation rather than direct evidence of contaminant removal. Moreover, the short exposure duration, the absence of final pH measurements, and the lack of direct measurements of photosynthetic activity, transpiration, and processes involved in OTC transport limit mechanistic interpretation of the observed responses. Future studies should incorporate longer-term exposures, environmentally realistic water matrices, measurements of OTC remaining in the aqueous phase, monitoring of pH throughout exposure, and additional physiological endpoints to determine the long-term tolerance of *B. laevis* and more directly evaluate its potential for OTC phytoremediation.

## Figures and Tables

**Table 1:** Concentrations, mass, and distribution of OTC in the organs of *B. laevis* *.

Treatments (µg L^−1^)	Concentration Shoots (µg kg^−1^ fw)	Concentration Roots (µg kg^−1^ fw)	Total Concentration (µg kg^−1^ fw)	Mass Shoot (µg)	Mass Root (µg)	Total Mass (µg)	Distribution Shoots (%)	Distribution Roots (%)
0.05	102.67 ± 22.3 A ab	32 ± 22.62 A a	74.4 ± 43.3 a	0.72 ± 0.13 A a	0.14 ± 0.1 A a	0.48 ± 0.34 a	84.39	15.61
0.5	77.33 ± 65.43 A a	80.5 ± 17.08 A a	75.6 ± 47.86 a	0.367 ± 0.3 A a	0.29 ± 0.07 A a	0.32 ± 0.19 a	54.65	45.35
5	104 ± 20.88 A ab	123 ± 24.36 A a	114.86 ± 23.35 a	0.62 ± 0.14 A a	0.69 ± 0.27 A a	0.66 ± 0.22 a	49.88	50.12
10	132 ± 20.3 A ab	162 ± 50.9 A a	144.86 ± 33.53 a	0.67 ± 0.17 A a	0.61 ± 0.27 A a	0.64 ± 0.18 a	48.73	51.27
100	165.33 ± 8.08 A b	931 ± 210.72 A b	471. 6 ± 432.44 b	0.89 ± 0.16 A a	3.77 ± 0.22 A b	2.04 ± 1.59 b	17.8	82.2

* Data of concentrations and mass are exhibited as mean ± SD (standard deviation). Similar uppercase letters indicate no significant differences between the roots and shoots from the same treatment (Wilcoxon rank-sum test, *p* ≥ 0.05), whereas similar lowercases indicates no significant differences between the treatments for each organ (one-way ANOVA, Tukey post hoc, *p* ≥ 0.05). In the case of total concentration and total mass, similar lowercases indicate no significant differences between the treatments (one-way ANOVA, Tukey post hoc, *p* ≥ 0.05).

**Table 2:** Bioaccumulation factor (BAF) for different organs and translocation factor (TF) between the shoot and roots for each treatment.

Treatment (µg L^−1^)	BAF Shoot (L kg^−1^)	BAF Root (L kg^−1^)	TF
0.05	5133.33	1600	3.21
0.5	166.31	173.12	0.96
5	21.89	25.89	0.85
10	13.31	16.33	0.81
100	1.58	8.91	0.18

## Data Availability

Data are contained within the article.

## Supplementary Materials

The following supporting information can be downloaded at: https://www.mdpi.com/article/10.3390/plants15182741/s1, Table S1: Partial least squares (PLS) regression coefficients relating OTC exposure (treatment) and organ OTC mass with the evaluated biomarkers and pigments in *B. laevis*. Positive coefficients indicate a positive association with the predictor, whereas negative coefficients indicate an inverse relationship; Table S2: Concentrations of oxytetracycline reported for Argentina and other countries in different matrices; Figure S1: (a–c) Representative images of *B. laevis* in its natural habitat in Pampean freshwater ecosystems, including streams and wetlands, and (d) germinated seed of *B*. *laevis* viewed under a stereomicroscope (4× magnification) obtained after mechanical scarification; Table S3: Dicotyledon nutrient solution composition for *B. laevis* hydroponic seedling growth; Table S4: Morphological measures of *B. laevis* after and before the OTC treatments. Data are represented as mean ± standard deviation (SD); Figure S2: Experimental workflow for the bioassay and oxytetracycline determination in *B. laevis*.
