# Supplementary material for: Oxytetracycline on the Wetland Macrophyte Bidens laevis L. Seedlings: Uptake, Bioaccumulation, Translocation and Biochemical Stress Responses
Source: Plants (Basel). 2026 Sep 8;15(18):2741. doi: 10.3390/plants15182741 (PMC13610351; doi:10.3390/plants15182741)
Supplement: Supplementary file 1 [file plants-15-02741-s001.zip › plants-4533860-supplementary.pdf]

Supplementary Material

Table S1. Partial least squares (PLS) regression coefficients relating OTC exposure (treatment) and organ OTC mass with the evaluated biomarkers and pigments in *B. laevis*. Positive coefficients indicate a positive association with the predictor, whereas negative coefficients indicate an inverse relationship.

Organ	Variable	Treatment	OTC mass	Main pattern
Shoot	CAT	+194.8	−1052.5	Exposure ↑; accumulation ↓
	GST	+1478.0	−5229.1	Exposure ↑; accumulation ↓
	GPOD	+1.21	−6.01	Exposure ↑; accumulation ↓
	SOD	+120.5	−934.0	Exposure ↑; accumulation ↓
	MDA	−0.00108	+0.00002	Exposure ↓; accumulation ↑
	H_2O_2	+0.00013	−0.00007	Weak response
	Chl *a*	+0.0277	+0.0586	Exposure ↑; accumulation ↑
	Chl *b*	+0.0144	+0.0366	Exposure ↑; accumulation ↑
	Chl total	+0.0421	+0.0952	Exposure ↑; accumulation ↑
	Chl a/b	+0.0317	−0.0630	Mixed pattern
Root	CAT	+1941	−1511	Exposure ↑; accumulation ↓
	GST	+7463	−6593	Exposure ↑; accumulation ↓
	GPOD	+37.6	−38.1	Exposure ↑; accumulation ↓
	SOD	+5727	−5166	Exposure ↑; accumulation ↓
	MDA	+0.00181	−0.00207	Exposure ↑; accumulation ↓
	H_2O_2	+0.00313	−0.00453	Exposure ↑; accumulation ↓

Table S2. Concentrations of oxytetracycline reported for Argentina and other countries in different matrices.

Site	Matrix	Average	SD	Max	Unit	Reference
Córdoba (Argentina)	Groundwaters	1.74	0.92	2.81	μg L^{-1}	[60]
	Groundwaters	2.44	1.21	5.29	μg L^{-1}	
	Groundwaters	2.17	0.86	3.95	μg L^{-1}	
	Groundwaters	2.25	1.33	5.29	μg L^{-1}	
	Groundwaters	2.25	0.82	1.93	μg L^{-1}	
	Groundwaters	2.2	1.38	1.8	μg L^{-1}	
	Groundwaters	1.74	0.92	2.81	μg L^{-1}	
	Groundwaters	2.44	1.21	5.29	μg L^{-1}	
	Groundwaters	2.17	0.86	3.95	μg L^{-1}	
	Groundwaters	2.25	1.33	5.29	μg L^{-1}	
	Groundwaters	2.25	0.82	1.93	μg L^{-1}	
Córdoba (Argentina)	Animal effluent	12		64	μg L^{-1}	[4]
Córdoba (Argentina)	*Oncorhynchus kisutch* (biota)	9.47		48.59	μg kg^{-1}	[61]
	O. mykiss (biota)	71.39		1151.25	μg kg^{-1}	
	Prochilodus lineatus (biota)	26.3		330.49	μg kg^{-1}	
	Piaractus mesopotamicus (biota)	5.16		15.57	μg kg^{-1}	
Buenos Aires (Argentina)	Sediments	1.9	0.4		mg kg^{-1}	[62]
Buenos Aires (Argentina)	Surface waters	11.8	0.2		mg L^{-1}	[63]
Buenos Aires (Argentina)	Sediments	39.8	8		mg kg^{-1}	[62]
Aquaculture systems in the north of Portugal	Surface water	11.9			ng L^{-1}	[64]
Fujian (China)	Surface water of a breeding farm	0.221	0.056		μg L^{-1}	[65]
Beijing (China)	Wastewater treatment plant	712 *	95		μg L^{-1}	[66]

*Highest mean recorded

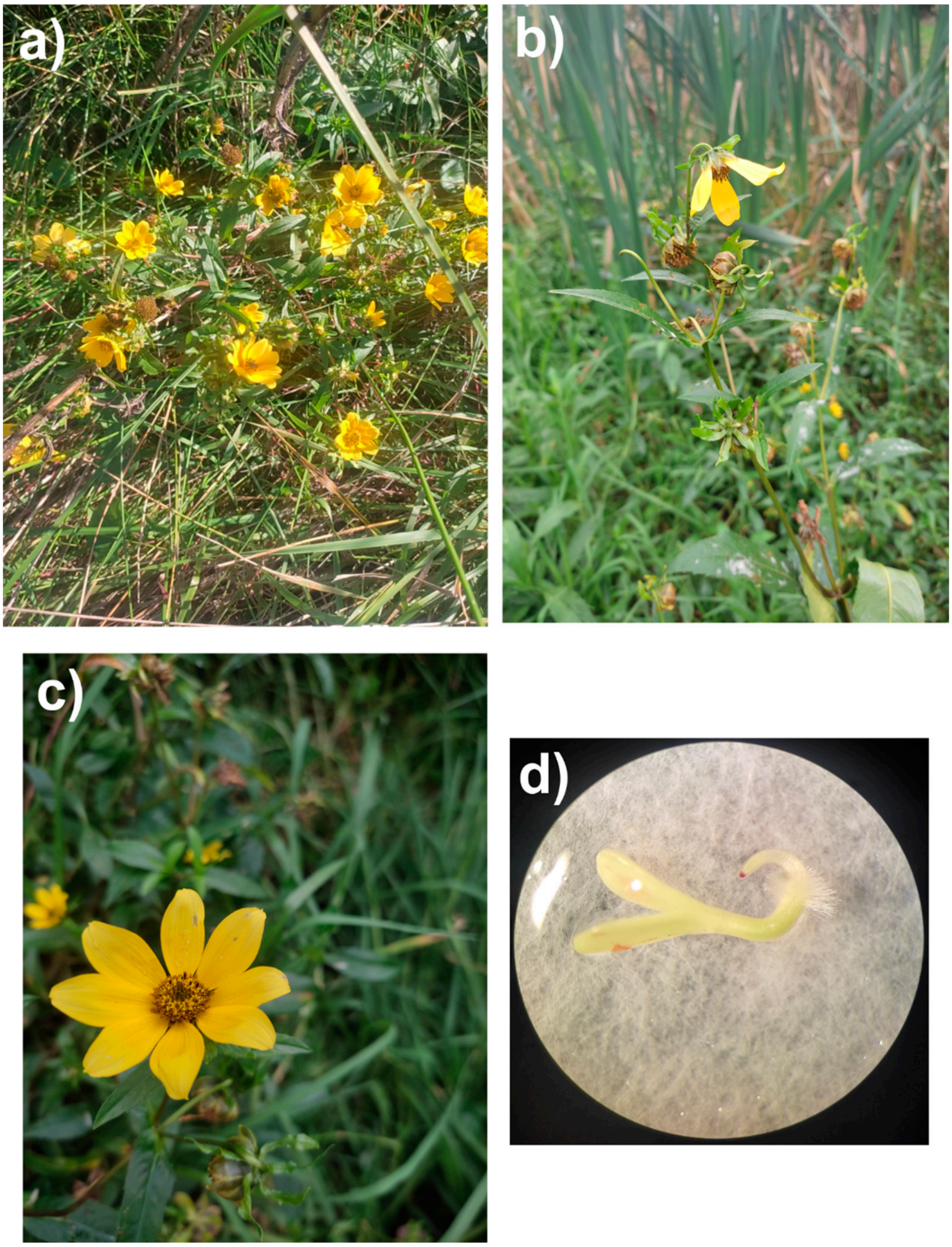

Figure S1. (**A, B, C**) Representative images of *B. laevis* in its natural habitat in Pampean freshwater ecosystems, including streams and wetlands, and (**D**) germinated seed of *B. laevis* viewed under a stereomicroscope (4× magnification) obtained after mechanical scarification.

Table S3. Dicotyledon nutrient solution composition for *B. laevis* hydroponic seedling growth*.

Stock solution	Component	Concentration
Nº1	$Ca\ (NO_3)_2$	524 mg L^{-1}
Nº2	KNO_3	221 mg L^{-1}
Nº3	$MgSO_4$	218 mg L^{-1}
Nº4	K_2HPO_4	62 mg L^{-1}
Nº5	H_3BO_3	1,1450 mg L^{-1}
	So_4Zn	0.088 mg L^{-1}
	$CuSO_4$	0.0316 mg L^{-1}
	$MnCl_2$	0.6840 mg L^{-1}
	Na_2MoO_4	0.0412 mg L^{-1}
Nº6	SO_4Fe_2	10 mg L^{-1}
	NaOH	3.98 mg L^{-1}

* A 100% Hoagland nutrient solution was prepared by adding 2.5 mL of each stock solution and adjusting the final volume to 1 L with distilled water.

Table S4. Morphological measures of *B. laevis* after and before the OTC treatments. Data is represented as mean ± standard deviation (SD)*.

Time (h)	Concentrations (µg L^{-1})	Shoot length (cm)	Root length (cm)	Total length (cm)	Total weight (g)
0	0	25.83 ± 1.6	24.73 ± 10.78	50.57 ± 11.3	9.8 ± 3.2
	0.05	28.17 ± 2.9	22.17 ± 4.79	50.33 ± 7.58	9.5 ± 1.1
	0.5	27.17 ± 3.87	24.83 ± 10.13	52 ± 12.82	8.19 ± 2.34
	5	26.33 ± 2.88	26.33 ± 8.55	52.67 ± 9.73	9.63 ± 2.34
	10	26 ± 2.45	22.17 ± 4.31	48.17 ± 3.97	8.55 ± 2.59
	100	24.5 ± 2.17	24.42 ± 7.06	48.92 ± 6.74	8.11 ± 1.67
48	0	30.17 ± 1.47	23.17 ± 10.13	53.33 ± 11.48	10.03 ± 2.79
	0.05	31.5 ± 4.04	19 ± 5.55	50.5 ± 9.44	11.07 ± 1.34
	0.5	30.58 ± 4.84	24.42 ± 9.58	55 ± 11.88	8.78 ± 2.41
	5	29.83 ± 2.4	25.83 ± 9.26	55.67 ± 10.8	10.83 ± 2.39
	10	28.83 ± 2.23	24 ± 5.1	52.83 ± 4.45	9.04 ± 2.8
	100	27.83 ± 1.72	22.33 ± 6.8	50.16 ± 5.74	9.3 ± 2.66

* No statistical differences were observed for the treatments over the 48-h of exposure.

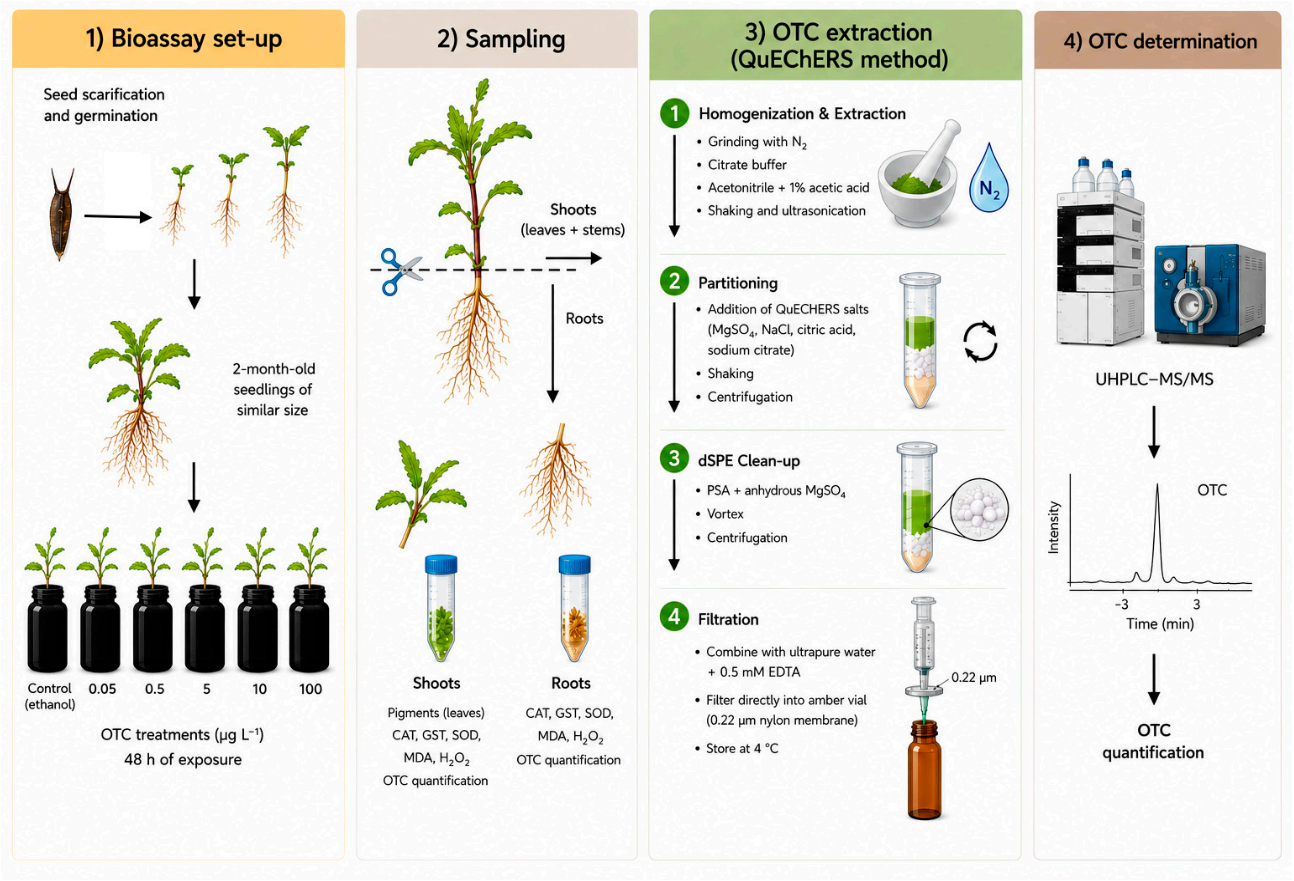

Figure S2. Experimental workflow for the bioassay and oxytetracycline determination in *B. laevis*.

Supplementary references

60. Borello, J.S.; Giannini-Kurina, F.; Grassi, D.A.; Tentor, G.; Cañas, A.I.; Nassetta, M.M.; Lepori, E.C.V. Veterinary drugs in groundwater in a dairy region of central Argentina and risk assessment for human health. *Environ. Qual. Manag.* **2023**, *33*, 239–255. https://doi.org/10.1002/tqem.22042.

61. Griboff, J.; Carrizo, J.C.; Bonansea, R.I.; Valdés, M.E.; Wunderlin, D.A.; Amé, M.V. Multiantibiotic residues in commercial fish from Argentina. The presence of mixtures of antibiotics in edible fish, a challenge to health risk assessment. *Food Chem.* **2020**, *332*, 127380. https://doi.org/10.1016/j.foodchem.2020.127380

62. Peluso, J.; Chehda, A.M.; Olivelli, M.S.; Ivanic, F.M.; Pérez Coll, C.S.; Gonzalez, F.; Valenzuela, L.; Rojas, D.; Cristos, D.; Butler, M.; Candal, R.J.; Aronzon, C.M. Metals, pesticides, and emerging contaminants on water bodies from agricultural areas and the effects on a native amphibian. *Environ. Res.* **2023**, *226*, 115692. https://doi.org/10.1016/j.envres.2023.115692.

63. Peluso, J.; Martínez Chehda, A.; Olivelli, M.S.; Ivanic, F.M.; Butler, M.; Aparicio, V.; de Geronimo, E.; Gonzalez, F.; Valenzuela, L.; Candal, R.J.; Aronzon, C.M. Impacts of cattle management and agricultural practices on water quality through different approaches: Physicochemical and ecotoxicological parameters. *Environ. Sci. Pollut. Res.* **2024**, *31*, 45177–45191. https://doi.org/10.1007/s11356-024-34059-2.

64. Pereira, A.M.P.T.; Silva, L.J.G.; Meisel, L.M.; Pena, A. Fluoroquinolones and tetracycline antibiotics in a Portuguese aquaculture system and aquatic surroundings: Occurrence and environmental impact. *J. Toxicol. Environ. Health A* **2015**, *78*, 959–975. https://doi.org/10.1080/15287394.2015.1036185.

65. Zhang, D.; Lin, L.; Luo, Z.; Yan, C.; Zhang, X. Occurrence of selected antibiotics in Jiulongjiang River in various seasons, South China. *J. Environ. Monit.* **2011**, *13*, 1953. https://doi.org/10.1039/c0em00765j.

66. Li, D.; Yang, M.; Hu, J.; Ren, L.; Zhang, Y.; Li, K. Determination and fate of oxytetracycline and related compounds in oxytetracycline production wastewater and the receiving river. *Environ. Toxicol. Chem.* **2008**, *27*, 80–86. https://doi.org/10.1897/07-080.1.
